# ArkDTA: attention regularization guided by non-covalent interactions for explainable drug–target binding affinity prediction

**DOI:** 10.1093/bioinformatics/btad207

**Published:** 2023-06-30

**Authors:** Mogan Gim, Junseok Choe, Seungheun Baek, Jueon Park, Chaeeun Lee, Minjae Ju, Sumin Lee, Jaewoo Kang

**Affiliations:** Department of Computer Science and Engineering, Korea University, Seoul 02841, Republic of Korea; Department of Computer Science and Engineering, Korea University, Seoul 02841, Republic of Korea; Department of Computer Science and Engineering, Korea University, Seoul 02841, Republic of Korea; Department of Computer Science and Engineering, Korea University, Seoul 02841, Republic of Korea; Department of Computer Science and Engineering, Korea University, Seoul 02841, Republic of Korea; LG CNS, AI Research Center, Seoul 07795, Republic of Korea; LG AI Research, Seoul 07795, Republic of Korea; Department of Computer Science and Engineering, Korea University, Seoul 02841, Republic of Korea; AIGEN Sciences, Seoul 04778, Republic of Korea

## Abstract

**Motivation:**

Protein–ligand binding affinity prediction is a central task in drug design and development. Cross-modal attention mechanism has recently become a core component of many deep learning models due to its potential to improve model explainability. Non-covalent interactions (NCIs), one of the most critical domain knowledge in binding affinity prediction task, should be incorporated into protein–ligand attention mechanism for more explainable deep drug–target interaction models. We propose ArkDTA, a novel deep neural architecture for explainable binding affinity prediction guided by NCIs.

**Results:**

Experimental results show that ArkDTA achieves predictive performance comparable to current state-of-the-art models while significantly improving model explainability. Qualitative investigation into our novel attention mechanism reveals that ArkDTA can identify potential regions for NCIs between candidate drug compounds and target proteins, as well as guiding internal operations of the model in a more interpretable and domain-aware manner.

**Availability:**

ArkDTA is available at https://github.com/dmis-lab/ArkDTA

**Contact:**

kangj@korea.ac.kr

## 1 Introduction

Identification of drug–target interactions (DTIs) is a central task in drug design and development. Due to the costly and labor-intensive nature of traditional drug development process based on *in vivo* and *in vitro* experiments, deep learning models for protein–ligand binding affinity prediction have gained recognition (Chen et al. 2018). However, limited model explainability remains an obstacle to the adoption of such models by domain experts (Preuer et al. 2019). With the unique ethical and regulatory requirements, there is a growing demand for interpretable deep models in the field of biomedicine. In recent works, attention-based methods were studied to address the issue of explainability (Liang et al. 2021). Critical domain knowledge should be integrated to ensure that the model’s implicit assumptions are compatible with expert opinions (Dash et al. 2022).

In DTI, one such key concept is that of protein–ligand non-covalent interactions (NCIs). NCIs are essential for understanding how proteins and ligands interact and form complexes with each other, which affects the mechanism of action for drug compounds (Tang et al. 2017; Chen et al. 2019; Anighoro 2020; Aljoundi et al. 2020). Most drug compounds are small organic molecules that act as ligands and interact with proteins to carry out their functions. The majority of drugs deliver their effects by forming noncovalent bonds with their biological targets. NCIs induce conformational changes in target proteins which influences the overall binding affinity. This is crucial for the stabilization of the protein–ligand complex in its final form (Davis and Phipps 2017; Aljoundi et al. 2020).

Despite being highlighted as a fundamental concept in protein–ligand affinity prediction task, few studies have addressed the importance of protein–ligand NCIs. While MONN (Li et al. 2020) explicitly utilized NCIs in its auxiliary task, the resulting pairwise interaction matrix between all protein residues and all ligand atoms is limited in its capacity to differentiate active and inactive binding sites. On the other hand, AttentionDTA sought to distinguish active and inactive residues using parameterized weights in their attention mechanism without explicitly using NCI labels. However, its attention mechanism is based on the convoluted features from each of its respective modality-wise encoder modules. We addressed the limitations of both previous works by utilizing NCI labels in the attention mechanism to identify active protein residues (Zhao et al. 2022a).

We present ArkDTA, an explainable deep DTI prediction model with NCI-aware attention regularization. Taking as input a set of protein residues and a set of ligand substructures, our novel regularization method modulates the distribution of cross-modal attention weights from the residues to chemical substructures in a manner that allows a distinction between active and inactive residues. Our modified cross-modal attention module appends a pseudo-substructure embedding to the set of key chemical substructures and focuses the attention on the pseudo embedding where the query protein residue is inactive. Examining the final attention weights yields qualitative insights into the model’s internal operations.

Experimental results on three benchmark datasets reveal that ArkDTA achieves predictive performance comparable to the current state-of-the-art models while significantly improving model explainability. Qualitative investigation into the attention maps demonstrates our model’s ability to identify NCI-forming regions in seen and unseen protein–ligand complexes as well as highlight chemical substructures commonly used as pharmaceutical agents.

## 2 Materials and methods

### 2.1 Dataset

Three different benchmark datasets were used in this study which are PDBbind version 2020 (PDBbind), Davis et al. (Davis), Metz et al. (Metz) to conduct experiments on *ArkDTA* and baseline models (Davis et al. 2011; Metz et al. 2011; Liu et al. 2017). The *i*th data instance $X_{i}$ in each of these datasets consists of a protein–ligand pair with its binding affinity score, expressed in one of the following measurement types: inhibition constant ($K_{i}$), dissociation constant ($K_{d}$) and inhibitory concentration 50 (IC_50_).

For the purpose of this study, the PDBbind was sub-divided into two subsets according to the binding affinity measurement type. The KIKD subset consists of all protein–ligand instances whose binding affinity scores are expressed as $K_{i}$ or $K_{d}$ value, and the remaining instances whose affinity scores are expressed as IC_50_ value were combined to form the IC_50_ subset. The Davis and Metz dataset contains protein–ligand pairs with only KIKD-based affinity scores.

We applied several data curation methods to our constructed datasets. For each dataset, protein–ligand pairs whose number of amino acids in the protein sequence exceeds 1000 or the exact affinity value is unavailable (e.g. expressed as inequality “>50000 M”) were excluded. We then normalized binding affinity scores in each dataset into values in unit “M” and subsequently transformed them into log space for consistent comparison (Öztürk et al. 2018). Table 1 shows the total number of proteins, ligands, and curated data instances in two measurement types (KIKD, IC_50_) for each dataset. Overall, each *i*th data instance $X_{i}$ in the binding affinity datasets is defined as the following, where $p_{i}$, $c_{i}$, $y_{i}\in R$ are input protein, ligand, and annotated binding affinity value, respectively. Table 1 shows the statistics of each dataset.


(1) $$X_{i}=(p_{i},c_{i},y_{i})$$

**Table 1.** Statistics of the DTI datasets used in experiments.

Dataset	PDBbind	Davis et al.	Metz et al.
No. of proteins	10 162	311	121
No. of ligands	13 015	68	240
No. of KIKD instances	9327	21 331	13 669
No. of IC_50_ instances	6 593	0	0

To regularize *ArkDTA*’s attention mechanism, we further augmented the preprocessed PDBbind dataset with NCI labels. We used Protein–Ligand Interaction Profiler (PLIP) to extract the NCI labels from each binding complex structure contained in the original PDBbind dataset (Adasme et al. 2021). The NCI labels for each protein–ligand pair are represented as a m×n 2D binary matrix to indicate the presence of any type of NCIs (e.g. hydrogen bonding, salt bridges) where *m* and *n* are the numbers of amino acid residues and atoms in ligand, respectively. Since the attention mechanism in *ArkDTA* is based on cross-modal interactions between the protein residues and chemical substructures, we converted the binary matrix into a *m*-dimensional binary vector $\vec{k}$ where each residue in protein is labeled as 1 if it has NCI with at least one atom in its ligand partner. We use $\vec{k}$ as ground truth residue-wise NCI labels for attention regularization in *ArkDTA*. The augmented *i*th data instance ${\overset{’}{X}}_{i}$ in the PDBbind dataset is defined as the following, where ${\vec{k}}_{i}\in {\left[0,1\right]}^{n}$ is the converted binary vector indicating the presence of each residue’s NCI with the input ligand $c_{i}$.


(2) $${\overset{’}{X}}_{i}=(p_{i},c_{i},y_{i},{\vec{k}}_{i})$$

Despite having the least data instances, our preprocessed PDBbind dataset is the primary dataset of this study since it contains the residue-wise NCI labels. We randomly partitioned the PDBbind dataset into 5-folds where 5% of the training instances in each fold were used for validation. This split method yields an ensemble of five models trained on different folds of this dataset. The Davis and Metz dataset used for fine-tuning purposes was partitioned into training and test instances (8:2) where 5% of the training instances were also used for validation. The purpose of these two datasets is to fine-tune each of the five models previously trained on the PDBbind dataset.

### 2.2 Model architecture

#### 2.2.1 Overview

**Figure 1.**
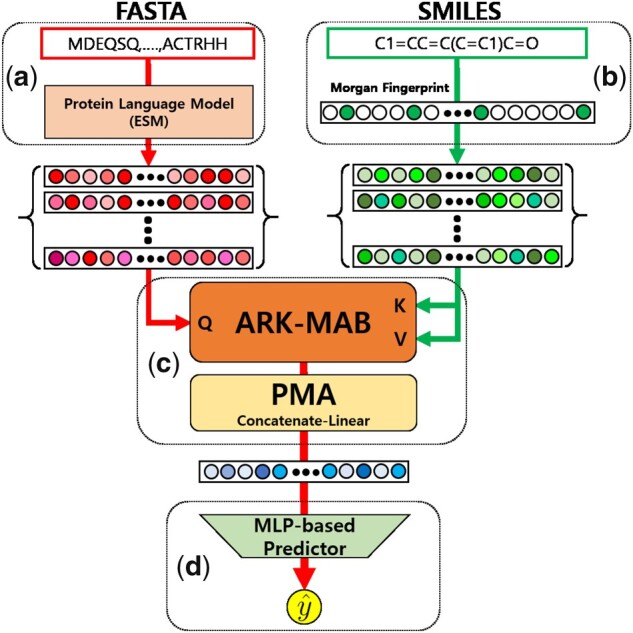
Overview of *ArkDTA*. **A** refers to the *Protein Encoder Module*, **B** refers to the *Ligand Encoder Module*, **C** refers to the *Protein*–*Ligand Integration Module* and **D** refers to the *Affinity Prediction Module*. $\hat{y}$ is the predicted binding affinity value.

Our *ArkDTA* model consists of ‘Protein Encoder Module’, ‘Ligand Encoder Module’, ‘Protein-Ligand Integration Module’, and ‘Affinity Prediction Module’. The first two modules encode input data into protein residue-wise and chemical substructure-wise representations, respectively. The ‘Protein-Ligand Integration Module’ refines the residue-wise representations based on their attention weights given the substructure-wise representations and subsequently aggregates them into a single binding complex representation. Finally, the ‘Affinity Prediction Module’ takes the binding complex representation as input and predicts the binding affinity value as output. The formal definition for *ArkDTA* is the following, where the input are protein p and ligand (compound) c, and output is the predicted binding affinity value $\hat{y}$. Figure 1 shows the overall model architecture of ArkDTA.


(3) $$\begin{array}{cc} \hat{y} & =\mathrm{ArkDTA}(p,c) \end{array}$$

#### 2.2.2 Protein Encoder Module

The ‘Protein Encoder Module’ takes a protein p as input and encodes it into a set of *m d*-dimensional residue embeddings as output. The initial representation for input protein is its FASTA sequence. While such 1D-based representations may have limitations in representing proteins, large-scale language models have been introduced to alleviate these issues (Rives et al. 2019). These models have shown promising results in protein structure and function prediction tasks. We imported a pre-trained protein language model called Evolutionary Scale Model (ESM) and its tokenizer to obtain residue embeddings (Rives et al. 2019; Lin et al. 2022). The tokenizer converts the input protein p into a sequence of tokens subsequently fed to the ESM model. Finally, the ESM model converts the input tokens to a set of protein residue embeddings $R\in R^{m\times d}$. The model version of its pretrained weights is ESM-2 (8M) where its number of layers is 6.

#### 2.2.3 Ligand Encoder Module

The ‘Ligand Encoder Module’ takes a ligand c as input and encodes it into a set of *n d*-dimensional substructure embeddings as output. The initial representation for input ligand is its SMILES string. The SMILES string is first converted into a Morgan fingerprint represented as a 1024-dimensional bit vector $\vec{f}\in {\left[0,1\right]}^{1024}$. Each bit position in the vector indicates the presence of its corresponding chemical substructure. The ‘Ligand Encoder Module’ leverages this information by gathering the positional indices of that vector where its bit is 1 and uses a lookup table to obtain a set of trainable *d*-dimensional chemical substructure embeddings $S=\left\{s_{1},s_{2}\ldots s_{n}|s_{i}\in S\right\}$ where *n* is the number of chemical substructures extracted from $\vec{f}$. S is a set of 1024 trainable chemical substructures where each of them corresponds to its bit position in the Morgan fingerprint.

#### 2.2.4 Protein–Ligand Integration Module

The ‘Protein-Ligand Integration Module’ consists of a Multihead Attention Block (MAB) and a Pooling Layer. The MAB refines an input set of protein residues R from ‘Protein Encoder Module’ based on our novel attention mechanism with another input set of chemical substructures S from ‘Ligand Encoder Module’. The Pooling Layer subsequently aggregates the refined residue-wise embeddings into one single binding complex embedding. The MAB’s operations reflect the conformational transitions proteins undergo when bound to a ligand, while the Pooling Layer’s output corresponds to the final protein–ligand complex that determines the binding affinity value.

The MAB in ‘Protein-Ligand Integration Module’ employs multihead attention mechanism and produces a set of ‘refined’ residues given R and S as inputs (Vaswani et al. 2017; Lee et al. 2019). Following the definitions made by previous works, the MAB takes R, S, and S as queries, keys, and values, respectively.

Let $A\in R^{m\times n}$ be the calculated attention weights between the query and key linear projections of R and S, respectively. For each *i*th residue (i∈{1,2,…,m}), the attention weights are distributed across all *n* corresponding chemical substructures where $\sum_{j=1}^{n}A_{i,j}=1$. However, as most residues do not form NCIs with the incoming ligand, it may be undesirable to utilize all calculated attention weights.

Our modified version of MAB first appends a trainable universal *d*-dimensional pseudo-substructure embedding $\vec{p}\in R^{d}$ to current set of chemical substructure embeddings S. The main purpose is to regularize the attention between the query protein residues and key-value chemical substructures based on their NCIs. Specifically, we devised a strategy that makes the attention weights from non-binding query residues (i.e. residues having no NCIs with ligand) skewed toward the key pseudo-substructure embedding. For binding query residues (i.e. residues having NCIs with ligand), the attention weights are prevented from being skewed toward the pseudo-substructure but distributed to actual chemical substructure embeddings in an unsupervised fashion. We denote this modification as Attention Regularization based on NCIs in MAB (ARK-MAB).

The ARK-MAB that takes R and S as input is mathematically expressed as follows, where $R^{*}\in R^{m\times d}$ is a set of ‘refined’ residue embeddings, ‘LayerNorm’ is layer-wise normalization method (Ba et al. 2016) and RFF is row-wise feedforward layer. *MultiAttn* is *k*-headed attention layer where X is linearly projected to query vectors and Y is linearly projected to both key and value vectors. For the calculation of attention weights, we employed Additive Attention originally proposed by Bahdanau et al. (2014) and used four attention heads.


(4) $$S^{+}=S\cup \left\{\vec{p}\right\}$$

(5) $$R^{*}=\mathrm{ARKMAB}(S^{+},R)$$

(6) $$\mathrm{ARKMAB}(S^{+},R)=\mathrm{LayerNorm}(H+RFF_{2}(R))$$

(7) $$H=\mathrm{LayerNorm}(R+RFF_{1}(\mathrm{MultiAttn}(R,S^{+},S^{+})))$$

We adopted Pooling by Multihead Attention (PMA) from the Set Transformer framework (Lee et al. 2019) for the Pooling Layer. The *m* refined residue embeddings $R^{*}\in R^{m\times d}$ are aggregated based on a set of *u* trainable seed vectors $U\in R^{u\times d}$ into a set of *u* aggregated residue embeddings $R^{a}\in R^{u\times d}$. Following the explanation in the Set Transformer paper, the PMA layer is built based on the MAB that takes U, $R^{*}$, and $R^{*}$ as queries, keys, and values, respectively. Subsequently, the aggregated residues are concatenated vector-wise and reduced to a *d*-dimensional binding complex embedding via a simple linear layer. The order of vector-wise concatenation is determined by the fixed order of seed vectors U.

**Figure 2.**
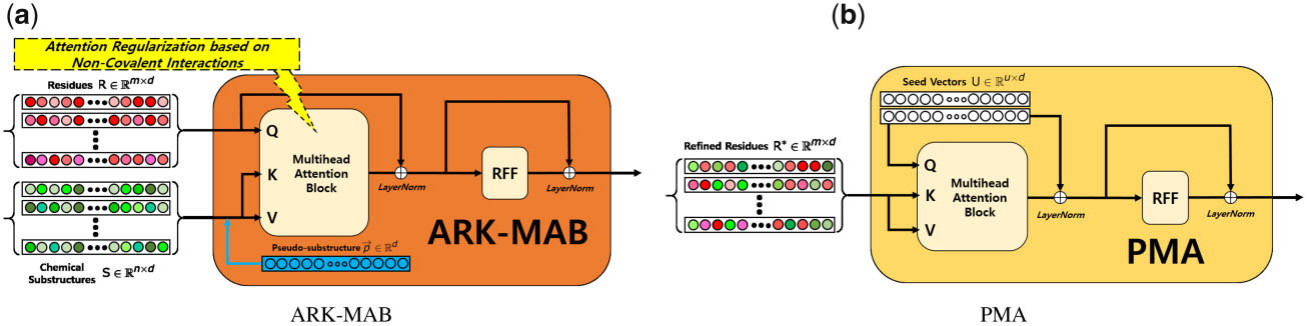
Detailed description of ARK-MAB (a) and PMA (b). (a) The input queries is a set of *m* residues while the key values is a set of *n* chemical substructures appended with a pseudo-embedding $\vec{p}$. The output of this sub-module is a set of *m* refined residues $R^{*}\in R^{m\times d}$. (b) The input queries is a set of *u* seed vectors while the key values is a set of *m* refined residues. The output of this sub-module is a set of *u* aggregated residues concatenated to each other. Note that the order of vector-wise concatenation is determined by the fixed order of seed vectors.

The PMA layer that takes the refined residues $R^{*}$ as input is mathematically expressed as follows, where $C\in R^{1\times d}$ is the binding complex embedding built from vector-wise concatenation (⊕) of the aggregated residues $\left\{r_{i}^{a}\in R^{a}|i=1,2,\ldots ,u\right\}$. Linear is linear layer without nonlinear activation that reduces the binding complex embedding’s expanded dimension to *d* where the weights and bias are $W_{\mathrm{linear}}\in R^{d\dot{u}\times d}$, $b_{\mathrm{linear}}\in R^{d}$, respectively. Figure 2 shows the detailed description of ARK-MAB and PMA.


(8) $$\begin{array}{c} R^{a}=PMA(R^{*}) \end{array}$$

(9) PMA(X)=MAB(U,X)

(10) $$\begin{array}{c} C=\mathrm{Linear}([r_{1}^{a}⊕\cdots ⊕r_{u}^{a}]) \end{array}$$

#### 2.2.5 Affinity Prediction Module

The ‘Affinity Prediction Module’ that takes the binding complex embedding C as input for predicting the binding affinity score $\hat{y}$ is mathematically expressed as follows, where $\hat{y}\in R$. $MLP_{\mathrm{score}}$ is a two-layered MLP where the intermediate layers use Dropout and ReLU as nonlinear activation. The weights, bias in the linear layers from top to bottom are $W_{\mathrm{score}1}\in R^{d\;\times d}$, $b_{\mathrm{score}1}\in R^{d}$, $W_{\mathrm{score}2}\in R^{d\times 1}$, $b_{\mathrm{score}2}\in R^{1}$, respectively.


(11) $$\begin{array}{c} \hat{y}=MLP_{\mathrm{score}}(C) \end{array}$$

#### 2.2.6 Training and optimization

The loss objective for training *ArkDTA* consists of two terms which are the main and auxiliary loss objective. The main loss objective is based on root mean squared error (RMSE) between the binding affinity predictions and each of their corresponding values. The auxiliary loss objective was specially designed to impose regularization on the attention mechanism utilized in the ‘Protein-Ligand Integration Module’ using binary cross entropy as its criterion.

The batch-wise main loss objective for binding affinity prediction is mathematically expressed as follows, where $\hat{Y}$ is a *b*-sized batch of predicted binding affinities, Y is a *b*-sized batch of ground truth binding affinities, and *MSE* is mean squared loss criterion for binding affinity prediction.


(12) $$\begin{array}{c} L_{1}(\hat{Y},Y)=\sqrt{\mathrm{MSE}(\hat{Y},Y)} \end{array}$$

(13) $$\hat{Y}=\left[\begin{array}{ccc} {\hat{y}}_{1} & \cdots & {\hat{y}}_{b} \end{array}\right]$$

(14) $$\begin{array}{c} Y=\left[\begin{array}{ccc} y_{1} & \cdots & y_{b} \end{array}\right] \end{array}$$

For attention regularization described in the ‘Protein-Ligand Integration Module’, let ${\bar{A}}^{+}\in R^{m\times (n+1)}$ be the calculated attention weight matrix averaged head-wise, given the set of *m* protein residue embeddings $R^{*}\in R^{m\times d}$ as queries and set of *n* chemical substructure embeddings $S\in R^{(n+1)\times d}$ appended with pseudo-embedding $\vec{p}$ as keys. For each residue in ${\bar{A}}^{+}$, the summation of *n* attention weights corresponding to *n* chemical substructures is equivalent to the NCI score deemed as the predicted class probability having NCI with the ligand compound. On the contrary, the attention weight corresponding to the pseudo-substructure is deemed as the predicted class probability having no such interactions.

**Figure 3.**
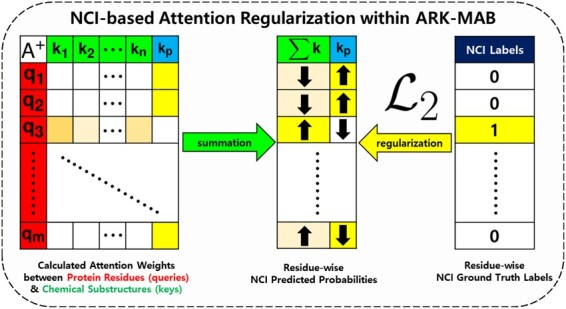
Schematic illustration of the ‘Protein-Ligand Integration Module’ in *ArkDTA* which features attention regularization based on NCIs between protein and ligand. The query and key projections of *m* residues R and *n* chemical substructures S appended with $\vec{p}$ are $\left\{q_{1},q_{2},\ldots ,q_{m}\right\}$ and $\left\{k_{1},k_{2},\ldots ,k_{n},k_{p}\right\}$, respectively. The auxiliary loss objective $L_{2}$ enforces *ArkDTA* to focus most of the attention toward $k_{p}$ when given a query residue without NCI. On the other hand, the $L_{2}$ encourages *ArkDTA* to only distribute its attention on actual chemical substructures from $k_{1}$ to $k_{n}$ in an unsupervised fashion.

Figure 3 illustrates how the attention mechanism in the ARK-MAB works. If the *i*th residue does not have any NCI with the ligand, the ARK-MAB is guided to generate attention weights ${\bar{A}}^{+}\in R^{m\times (n+1)}$ where $\sum_{j=1}^{n}{\bar{A}}_{i,j}<{\bar{A}}_{i,n+1}^{+}$. In other words, the *i*th residue is expected to be mostly attended against the pseudo-substructure $\vec{p}$ and relatively less attended to the actual chemical substructures. On the contrary, if the residue has an actual NCI, the guided attention weights are distributed to actual chemical substructures in an unsupervised fashion.

The batch-wise auxiliary loss objective for attention regularization is mathematically expressed as follows, where A is a *b*-sized batch of attention weight matrices averaged head-wise, K is a batch of ground truth NCI labels, ${\bar{A_{i}}}^{+}$ is the *i*th attention matrix where number of rows and columns are $m_{i}$ and $n_{i+1}$ respectively, $\hat{k}$ are the residue-wise predicted NCI probabilities based on column-wise summation of ${\bar{A_{i}}}^{+}$ except the last $n_{i}+1$th column which corresponds to the pseudo-substructure $\vec{p}$, $\hat{K}$ is a batch of predicted NCI probabilities, and ‘CrossEntropy’ is the binary cross entropy loss criterion for attention regularization based on NCI. For the *i*th instance in batch, $m_{i}$ and $n_{i}$ are the number of residues in protein and substructures in ligand, respectively. Recall that $\vec{k}$ is a *m*-dimensional binary vector where each bit indicates the corresponding residue having a NCI with its ligand partner. Supplementary Figure S3 provides an illustrative description for the auxiliary loss objective $L_{2}$.


(15) $$\begin{array}{c} L_{2}(A,K)=\mathrm{CrossEntropy}(\hat{K},K) \end{array}$$

(16) $$A=\left[\begin{array}{ccc} {\bar{A}}_{1}^{+} & \cdots & {\bar{A}}_{b}^{+} \end{array}\right]$$

(17) $$\begin{array}{c} {\bar{A_{i}}}^{+}={\left(a_{rc}\right)}_{1\leq r\leq m_{i},1\leq c\leq n_{i}+1} \end{array}$$

(18) $$\hat{k_{i}}=\sum_{c=1}^{n_{i}}{\bar{A}}_{i}^{+}\;(\sum_{c=1}^{n_{i}+1}{\bar{A}}_{i}^{+}=1)$$

(19) $$\begin{array}{c} \hat{K}=\left[\begin{array}{ccc} {\hat{\text{k}}}_{1} & \cdots & {\hat{\text{k}}}_{b} \end{array}\right] \end{array}$$

(20) $$K=\left[\begin{array}{ccc} {\vec{k}}_{1} & \cdots & {\vec{k}}_{b} \end{array}\right]$$

Overall, the total loss objective for training *ArkDTA* is mathematically expressed as follows, where L is a sum of two loss objectives. α is the NCI-based auxiliary loss coefficient that determines the intensity of guiding cross-modal attention mechanism in *ArkDTA*’s ARK-MAB.


(21) $$\begin{array}{c} L=L_{1}+\alpha L_{2} \end{array}$$

The base dimension size for all embeddings is set to d=320 while the number of trainable seed vectors U is 2. All *ArkDTA* and its ablations were trained to a maximum of 100 epochs with batch size of 64 and early stopping. The hyperparameters including learning rate and auxiliary loss coefficient α for *ArkDTA* were determined by its prediction performance on validation instances. We used the Adam optimizer with weight decay 0.0 for training *ArkDTA* on the binding affinity datasets. The learning rate was set to 0.00005 and 0.0001, while the auxiliary loss coefficient α was set to 5.0 and 1.0 for the KIKD-based (PDBbind KIKD Subset, Davis, Metz) and IC_50_-based (PDBbind IC50 Subset) datasets, respectively.

## 3 Results

### 3.1 Experiment settings

We trained *ArkDTA* on each subset (KIKD and IC_50_) of the PDBbind dataset which contains the NCI labels necessary for regularizing *ArkDTA*’s drug–target cross-modality attention mechanism. Since the data partition method is based on 5-fold cross-validation, we trained *ArkDTA* on each fold’s training instances and subsequently evaluated its binding affinity prediction performance on the corresponding test instances (**Test PDBbind—KIKD**). The evaluation metrics used in the experiments are RMSE, mean absolute error (MAE), Pearson’s correlation (PCORR), and concordance index (CI). All evaluation metrics were calculated using the mean and standard deviation of the five folds.

In addition, we gathered each fold’s test instances where each of its drug compound’s scaffold is not present in any of the other drug compounds of the training partition. A scaffold is a molecular core structure of a drug compound that determines its overall biochemical activity and is essential in drug design. We extracted each ligand’s Bemis–Murcko scaffold via the RDKit library (Bemis and Murcko 1996). If the input ligand in a test data instance has a molecular scaffold that does not overlap with those of all training ligands, we deemed it as a test instance with unseen scaffold. To further evaluate *ArkDTA*’s robustness on compounds with unseen scaffolds, we additionally calculated the evaluation metrics on such data instances (**Test PDBbind—Unseen Scaffolds**).

We then loaded each of its model checkpoint to conduct additional experiments on the Davis and Metz dataset. By means of transferring NCI-related knowledge, we fine-tuned each of the models previously trained on the PDBbind dataset and evaluated them on the same test instances of the Davis and Metz dataset. The evaluation metrics were calculated based on the mean and standard deviation of five sets of individual model performances. Since only the KIKD-based instances are present in the Davis and Metz dataset, we evaluated its performance on only KIKD-based affinity predictions. The same experimental setting was applied to *ArkDTA*’s baselines and ablations as well.

### 3.2 Baseline models and *ArkDTA* ablations

The binding affinity prediction models used as baselines are the following: DeepDTA (Öztürk et al. 2018), GraphDTA (Nguyen et al. 2021), TransformerCPI (Chen et al. 2020), MONN (Li et al. 2020), HyperAttentionDTI (Zhao et al. 2022b), AttentionDTA (Zhao et al. 2022a), BACPI (Li et al. 2022), and IIFDTI (Cheng et al. 2022). Details on each of the baseline models can be found in Supplementary Table S1. Among those that employed cross-modality attention, only MONN and *ArkDTA* explicitly utilized the NCI labels to improve this mechanism. While MONN introduced a secondary downstream task for predicting ‘atom-residue pairwise’ NCIs, our model alternatively used ‘residue-wise’ NCIs by means of attention regularization since its ligand representation is based on set of chemical substructures.

The model hyperparameters and implementation were imported from each of their original works. Note that some baseline models were originally implemented to predict binary interaction outcomes instead of continuous binding affinity values. To circumvent this issue, we replaced the downstream classifier layers with the regression ones for TransformerCPI, HyperAttentionDTI, and IIFDTI.

To investigate the effects of our model design choices, we made the following model ablations for *ArkDTA*,

- **Remove** $L_{2}$ **:** We removed the auxiliary loss objective $L_{2}$ by setting the loss coefficient α to 0 which leaves the model being solely trained on the main binding affinity prediction task ($L=L_{1}$). The purpose of this ablation is to probe the effects of attention regularization given the residue-wise NCI labels as ground truths.
- **Freeze ESM**: We froze the pre-trained weights of the imported ESM model used in *ArkDTA*’s ‘Protein Encoder Module’. The purpose of this ablation is to investigate the effects of fine-tuning which is a common practice in optimizing large-scale language models on other downstream tasks.

### 3.3 Binding affinity prediction results

Table 2 shows the 5-fold cross-validation results on the PDBbind dataset. For the KIKD subset of the PDBbind dataset, *ArkDTA* performed slightly better than its ablated version without NCI-based auxiliary loss but fell behind one of the baseline models AttentionDTA in all evaluation metrics including the test instances with unseen scaffolds. For the IC_50_ subset of the PDBbind dataset, *ArkDTA* showed best performance compared to its ablations and baseline models except RMSE and MAE for all test instances.

**Table 2.** Cross-validation results on the PDBbind dataset.

Model	PDBbind KIKD				PDBbind KIKD (Unseen Scaffolds)				PDBbind IC50				PDBbind IC50 (Unseen Scaffolds)			
	RMSE	MAE	PCORR	CI	RMSE	MAE	PCORR	CI	RMSE	MAE	PCORR	CI	RMSE	MAE	PCORR	CI
ArkDTA (ours)	1.2941 (0.0673)	0.9618 (0.0375)	0.7671 (0.0194)	0.7898 (0.0073)	1.3452 (0.0734)	1.0231 (0.0440)	0.7362 (0.0227)	0.7717 (0.0108)	1.2073 (0.0522)	0.8536 (0.0288)	**0.7752 (0.0169)**	**0.7366 (0.0040)**	**1.1689 (0.0586)**	**0.8368 (0.0340)**	**0.7406 (0.0249)**	**0.7122 (0.0119)**
ArkDTA (Remove $L_{2}$)	1.3005 (0.0650)	0.9738 (0.0459)	0.7656 (0.0210)	0.7887 (0.0089)	1.3561 (0.0654)	1.0413 (0.0468)	0.7335 (0.0169)	0.7700 (0.0074)	**1.1931 (0.0360)**	0.8667 (0.0330)	0.7791 (0.0118)	0.7368 (0.0104)	1.1899 (0.0564)	0.8800 (0.0463)	0.7306 (0.0273)	0.7055 (0.0226)
ArkDTA (Freeze ESM)	1.3457 (0.0396)	1.0170 (0.0222)	0.7512 (0.0089)	0.7806 (0.0024)	1.4059 (0.0645)	1.0905 (0.0446)	0.7147 (0.0215)	0.7601 (0.0086)	1.2561 (0.0415)	0.9150 (0.0297)	0.7540 (0.0165)	0.7251 (0.0065)	1.2401 (0.0326)	0.9114 (0.0257)	0.7066 (0.0146)	0.6965 (0.0092)
DeepDTA	1.3589 (0.0534)	0.9761 (0.0405)	0.7657 (0.0145)	0.7879 (0.0077)	1.3651 (0.0338)	1.0354 (0.0517)	0.7278 (0.0225)	0.7687 (0.0119)	1.2131 (0.0468)	0.8794 (0.0291)	0.7687 (0.0259)	0.7349 (0.0124)	1.1963 (0.0655)	0.8721 (0.0439)	0.7195 (0.0453)	0.7071 (0.0160)
GraphDTA	1.5511 (0.0401)	1.1624 (0.0371)	0.6641 (0.0123)	0.7396 (0.0057)	1.5934 (0.0579)	1.2085 (0.0305)	0.6268 (0.0271)	0.7234 (0.0104)	1.5515 (0.1243)	1.1449 (0.1156)	0.6557 (0.0432)	0.6967 (0.0177)	1.5438 (0.1372)	1.1442 (0.1026)	0.5969 (0.0577)	0.6729 (0.0208)
TransformerCPI	1.4982 (0.0375)	1.1478 (0.0256)	0.6716 (0.0171)	0.7455 (0.0079)	1.5008 (0.0551)	1.1606 (0.0356)	0.6519 (0.0236)	0.7348 (0.0090)	1.3465 (0.0332)	0.9646 (0.0373)	0.7190 (0.0120)	0.7144 (0.0083)	1.2905 (0.0761)	0.9332 (0.0538)	0.6816 (0.0399)	0.6931 (0.0164)
MONN	1.3283 (0.0440)	0.9927 (0.0193)	0.7563 (0.0137)	0.7849 (0.0061)	1.3534 (0.0822)	1.0288 (0.0482)	0.7343 (0.0264)	0.7736 (0.0105)	1.3994 (0.0417)	1.0450 (0.0290)	0.6725 (0.0286)	0.6936 (0.0131)	1.3242 (0.0388)	0.9749 (0.0171)	0.6414 (0.0270)	0.6732 (0.0113)
HyperAttentionDTI	1.4028 (0.0186)	1.0888 (0.0192)	0.7257 (0.0149)	0.7683 (0.0061)	1.4480 (0.0418)	1.1399 (0.0351)	0.6899 (0.0153)	0.7510 (0.0067)	1.2994 (0.0293)	0.9816 (0.0265)	0.7371 (0.0142)	0.7233 (0.0047)	1.2652 (0.0406)	0.9594 (0.0332)	0.6879 (0.0295)	0.6962 (0.0049)
AttentionDTA	**1.2711 (0.0454)**	**0.9410 (0.0297)**	**0.7726 (0.0125)**	**0.7908 (0.0063)**	**1.3213 (0.0500)**	**1.0074 (0.0341)**	**0.7372 (0.0169)**	**0.7716 (0.0063)**	1.2131 (0.0639)	**0.8420 (0.0425)**	0.7721 (0.0254)	0.7446 (0.0136)	1.2071 (0.0712)	0.8456 (0.0437)	0.7211 (0.0369)	0.7151 (0.0171)
BACPI	1.3594 (0.0362)	1.0164 (0.0152)	0.7427 (0.0139)	0.7772 (0.0055)	1.4343 (0.0660)	1.0998 (0.0380)	0.6974 (0.0320)	0.7545 (0.0116)	1.3583 (0.0577)	1.0439 (0.0395)	0.7140 (0.0285)	0.7278 (0.0152)	1.3798 (0.0652)	1.0702 (0.0505)	0.6507 (0.0385)	0.7001 (0.0168)
IIFDTI	1.3832 (0.0590)	1.0513 (0.0594)	0.7332 (0.0242)	0.7701 (0.0123)	1.4340 (0.0817)	1.1169 (0.0662)	0.6891 (0.0383)	0.7474 (0.0166)	1.3027 (0.0554)	0.9326 (0.0436)	0.7345 (0.0235)	0.7116 (0.0173)	1.2868 (0.0625)	0.9261 (0.0446)	0.6774 (0.0380)	0.6827 (0.0179)

Main cross-validation results for *ArkDTA*’s performance binding affinity prediction compared with its baselines and ablations. The results for both KIKD and IC_50_ subsets of the PDBbind dataset are based on mean and standard deviation of its five different test folds. **Unseen Scaffolds** refers to model’s performance evaluated on test instances where the ligands’ compound scaffolds do not overlap with those of the training instances in each fold. Best performance values are shown in bold.

Table 3 shows the additional results on the Davis and Metz dataset. Among the baselines, we selected the top four performing models DeepDTA, MONN, AttentionDTA, and BACPI based on their performance in the KIKD subset of the PDBbind dataset. Among the five models, AttentionDTA overall showed best performance in both the Davis and Metz dataset.

**Table 3.** Additional results on the Davis and Metz dataset

Model	Davis KIKD				Metz KIKD			
	RMSE	MAE	PCORR	CI	RMSE	MAE	PCORR	CI
ArkDTA (ours)	0.4979 (0.0132)	0.2863 (0.0105)	0.8176 (0.0104)	0.8684 (0.0053)	0.4127 (0.0066)	0.2559 (0.0063)	0.8336 (0.0049)	0.8430 (0.0034)
ArkDTA (Remove $L_{2}$)	0.4959 (0.0071)	0.3041 (0.0072)	0.8193 (0.0061)	0.8647 (0.0034)	0.4177 (0.0099)	0.2748 (0.0084)	0.8299 (0.0085)	0.8240 (0.0079)
DeepDTA	0.4753 (0.0061)	0.2548 (0.0134)	0.8367 (0.0054)	0.8737 (0.0082)	0.4049 (0.0069)	0.2376 (0.0055)	0.8416 (0.0042)	0.8588 (0.0064)
MONN	0.5217 (0.0065)	0.3240 (0.0075)	0.7993 (0.0063)	0.8621 (0.0044)	0.4619 (0.0102)	0.3050 (0.0134)	0.7853 (0.0115)	0.8084 (0.0077)
AttentionDTA	0.4927 (0.0137)	0.2466 (0.0129)	0.8230 (0.0106)	0.8696 (0.0063)	0.3944 (0.0177)	0.2150 (0.0161)	0.8491 (0.0137)	0.8737 (0.0106)

The results are based on mean and standard deviation of its five different model’s performance on the same test instances.

### 3.4 Analysis on attention weights

For qualitative analysis, we performed model inference and visualized the attention weights using heatmaps and compared them with actual binding complexes and ligand compound structures obtained from the Protein Data Bank (PDB) and PubChem database (Burley et al. 2023; Kim et al. 2023).

Given a protein–ligand pair input represented as a set of *m* residues and *n* chemical substructures respectively, the attention weights between *m* protein residues (queries) and *n* chemical substructures appended with a pseudo-substructure (keys) in each head are represented as a 2D matrix ${\bar{A}}^{+}$. We calculated the NCI score for each protein residue as shown in Equations (17) and (18). Since proteins are generally long sequences, we transposed the 2D matrix and truncated residue regions where both NCI scores and labels are deemed negative (i.e. no NCI between the residue and ligand).

**Figure 4.**
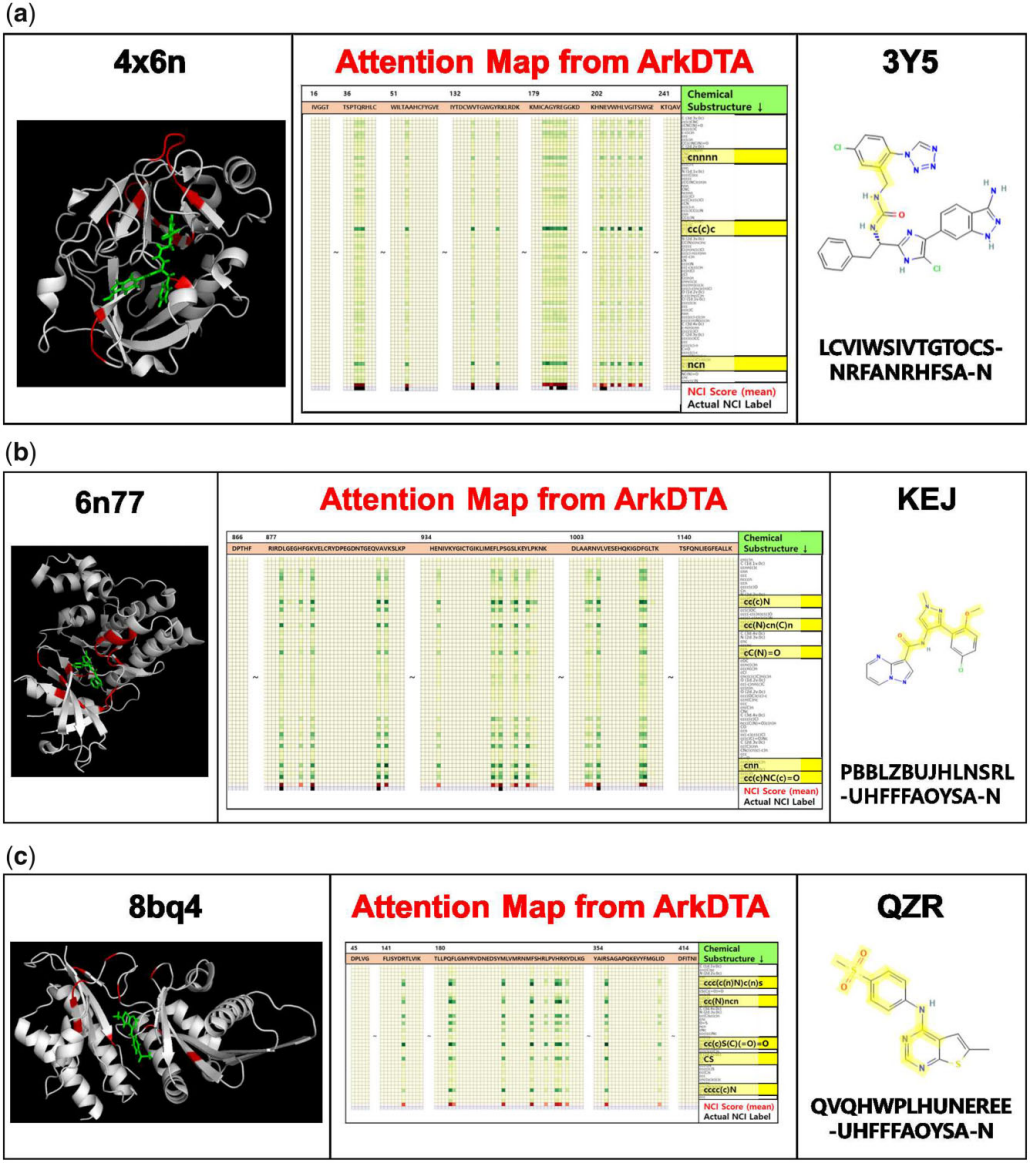
Visualization results on three protein–ligand pair examples. The left side shows a high-resolution 3D image of the co-crystallized binding complex structure obtained from its pdb file format using PyMol (Schrödinger and DeLano 2020). The right side shows the molecular structure of the ligand with its RCSB ligand identifier on the top and InChIKey on the bottom. The center shows the attention map extracted from *ArkDTA*. We truncated the amino residues whose NCI score suggests no NCI present and actual NCI label is negative. On the contrary, the amino acid residues identified as having NCIs (red-colored cells) correspond to the red-colored regions of the protein structure on the left side. The highlighted substructure SMILES (darker green-colored cells) correspond to the highlighted regions of the whole molecular structure on the right side. Full-sized attention maps for each cases can be found in the supplementary material. (a) Seen Protein & Unseen Ligand Binding Complex (**4x6n, 3Y5**), (b) Unseen Protein–Ligand Binding Complex (**6n77, KEJ**), and (c) Out-of-Dataset Binding Complex (**8bq4, QZR**).

As shown in Fig. 4a, the protein–ligand binding complex (Schrödinger and DeLano 2020) and molecular structure of the ligand are the left and right side, respectively. The following details that describe the attention map displayed on the center side are the following.

- The *m* columns correspond to the *m*-sized amino acid sequence of the input protein.
- The first *n* rows correspond to the chemical substructures extracted from the input ligand’s Morgan Fingerprint. Darker green colors indicate higher attention weights.
- The cell values in the row denoted as **NCI Score** are the sum of all attention weights distributed from each residue to all ligand’s chemical substructures. Darker red colors indicate higher NCI scores.
- The cell values in the row denoted as **Actual NCI Label** are the binary values indicating the actual presence of an NCI in each residue (black).

After obtaining the attention maps and related visualizations, we performed three different case studies.

#### 3.4.1 Seen protein & unseen ligand binding complex (4x6n, 3Y5)

In this case study, we selected an input protein contained in both training and test partition of the PDBBind dataset and a ligand that is only in its test partition. Figure 4a shows the visualization results performed on a binding complex structure (**4x6n**) of factor XIa with the inhibitor 1-{(1S)-1-[4-(3-amino-1H-indazol-6-yl)-5-chloro-1H-imidazol-2-yl]-2-phenylethyl}-3-[5-chloro-2-(1H-tetrazol-1-yl)benzyl]urea. Based on comparison between the calculated residue-wise NCI scores and its actual ground truth NCI labels, *ArkDTA* was able to not only identify the NCI positive residues but also identify their local regions as well. The highlighted areas of the protein–ligand binding complex structure also align with actual residue sites that seem to bind the incoming ligand.

#### 3.4.2 Unseen protein–ligand binding complex (6n77, KEJ)

In this case study, we selected a protein–ligand pair that is only in the test partition of PDBbind dataset. Figure 4b shows the visualization results performed on a binding complex structure of the JAK1 kinase domain (**6n77**) with the inhibitor ligand N-[3-(5-chloro-2-methoxyphenyl)-1-methyl-1H-pyrazol-4-yl]pyrazolo[1,5-a]pyrimidine-3-carboxamide (**KEJ**). In this case, despite the test instance being a unseen protein–ligand pair, the protein residues predicted as having NCIs based on their attention scores seemed to form plausible binding pockets for the ligand. Notably, *ArkDTA* highlighted the cyclic regions containing pyrazole-based substructures (cC(N)cn(C)n) which are renowned as important pharmacological scaffolds (Karrouchi et al. 2018).

#### 3.4.3 Out-of-dataset binding complex (8bq4, QZR)

Figure 4c shows the visualization results performed on a crystal binding complex structure (**8bq4**) of therapeutic target phosphatidylinositol 5-phosphate 4-kinases (PI5P4Ks) with the inhibitor ligand 6-methyl-N-(4-methylsulfonylphenyl)thieno[2,3-d]pyrimidin-4-amine (**QZR**) (Rooney et al. 2022). We examined the attention map to see whether *ArkDTA* is able to identify potential binding residues of an out-of-dataset drug–target pair. Interestingly, some of the residues in PI5PAks identified as having NCIs were located near the ligand. The surrounding residue sites may act as guidelines for generating grids prior to docking simulation. This demonstrates that *ArkDTA* has developed its own understanding on active protein residues due to the NCI-based attention regularization technique. One of the most highlighted substructures in the ligand is the sulfonyl functional group (cc(c)S(C)(=O)=O), which is commonly used in synthesizing drug compounds (Feng et al. 2016). Another highlighted substructure is related to pyrimidine (cc(N)ncn), a therapeutic scaffold which has various biological roles such as antiviral and antimalarial agent Kumar and Narasimhan (2018).

#### 3.4.4 Attention map comparison between ArkDTA and without $L_{2}$

Supplementary Figure S4 shows a comparison between *ArkDTA* and its ablated version of not employing NCI-based attention regularization. As shown in the ablated verion’s attention map, all protein residues are treated as having NCIs with the ligand. While this visualization may inform researchers with significant chemical substructures, it has limited explainability on the protein side. This highlights the role of NCIs in attention regularization which includes providing more salient information of the binding complex leading to better explainability. (Full-sized attention maps for the three case studies are available in Supplementary Fig. S5. Additional analysis on attention weights extracted from other protein-ligand complexes for each of the case studies are available in Supplementary Fig. S6.)

## 4 Discussion

### 4.1 Effect of attention regularization guided by NCIs

Preliminary statistical analysis shown in Supplementary Fig. S2 indicates that there is no obvious correlation between the ratio of active and inactive residues in a given protein and the binding affinity value of the protein–ligand complex. This suggests that our model’s ability to incorporate key domain knowledge such as NCIs in inference may not translate directly to improvements in binding affinity predictive performance. Nonetheless, our model was one of the three highest performing models in all evaluation metrics in above reported experimental setups, maintaining robust performance while significantly improving model interpretability. The auxiliary loss objective $L_{2}$ guides our model in identifying residues participating in NCIs with ligand substructures, distributing attention weights in a differentiated manner. The resulting attention maps and weights can be further investigated in order to gain insights on the potential interaction sites between newly designed candidate drugs and novel target proteins.

### 4.2 Limitations of ArkDTA and future work

A simplifying assumption for the representation of NCIs was that binary values indicating presence or absence of NCIs would provide sufficient information on the underlying chemical system. However, NCIs are typically sub-categorized according to characteristics such as their geometrical configurations, interaction strengths, and the kind of chemical force involved. Future works can take into account how different types of NCIs affect the overall binding behavior as proposed by Choe et al. (2022).

Another potential limitation in our work is the restrictive size of the training dataset. PDBbind is unique among publicly available datasets in that it provides coordinate data for each protein–ligand complex, which can be used to obtain NCI markers using tools such as PLIP (Adasme et al. 2021). However, PDBbind suffers a sparsity problem, containing binding affinity values for only a limited number of protein–ligand pairs relative to other benchmark datasets such as Davis and KIBA (Pahikkala et al. 2015; He et al. 2017). In future works, adoption of transfer learning and data augmentation methods can be explored to address this issue.

Despite the promising results from qualitative analysis, ArkDTA’s performance in four quantitative evaluation metrics does not fully capture the benefits of our NCI-aware regularization method in terms of model generalizability. We speculate that this is partly due to the absence of dedicated multi-objective loss function and optimization technique. In future work, we plan to design a multi-objective loss function and optimizer such that the relative weight given to each loss objective can be determined to minimize the potential antagonism between two loss objectives. In addition, we will investigate how changing the auxiliary loss coefficient α affects the model’s performance on the binding affinity prediction task.

## 5 Conclusion

In this work, we introduce *ArkDTA*, a protein–ligand binding affinity prediction model that employs a novel attention regularization technique guided by NCIs. While there is still room for improvements in the predictive performance, our model achieves significant improvements in model explainability over existing models. Furthermore, we found upon qualitative analysis of attention maps that the final distribution of attention weights can be used to gain insights into the model’s internal understanding of the underlying chemical system as well as suggest protein residues and chemical substructures of high pharmaceutical relevance.

## Supplementary Material

btad207_Supplementary_Data

## Data Availability

Our study used open-access datasets, and the data-related links are available in the github repository Which is mentioned above.
