# Supplementary material for: ArkDTA: attention regularization guided by non-covalent interactions for explainable drug–target binding affinity prediction
Source: Bioinformatics. 2023 Jun 30;39(Suppl 1):i448–57. doi: 10.1093/bioinformatics/btad207 (PMC10311339; doi:10.1093/bioinformatics/btad207)
Supplement: btad207_Supplementary_Data [file btad207_supplementary_data.docx]

Supplementary Data for ArkDTA: *Attention Regularization guided by non-Covalent Interactions for Explainable Drug-Target Binding Affinity Prediction*

**1. Baseline models used in experiments**


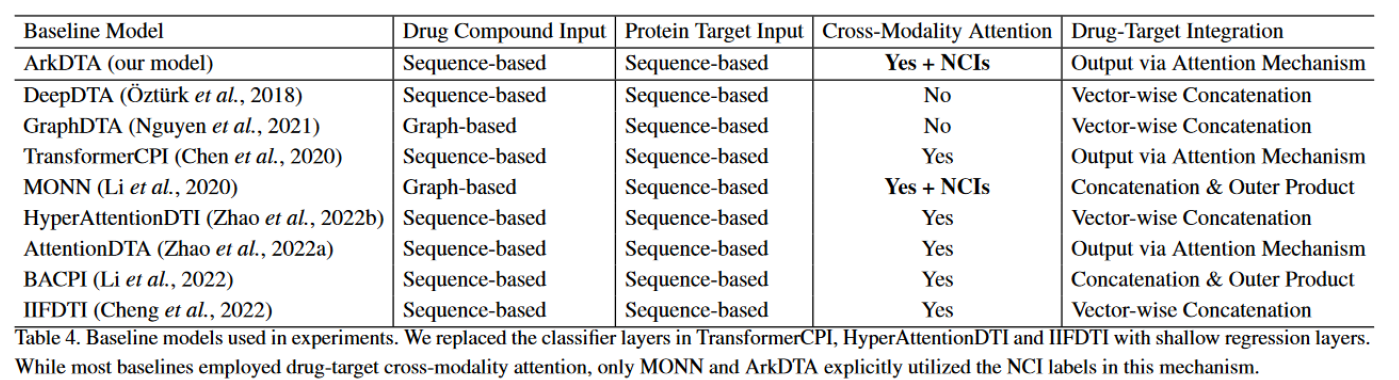


Table S1. Baseline models used in experiments. We replaced the classifier layers in TransformerCPI, HyperAttentionDTI and IIFDTI with shallow regression layers. While most baselines employed drug-target cross-modality attention, only MONN and ArkDTA explicitly utilized the NCI labels in this mechanism.

**2. Statistics related to the residue-wise NCI labels contained in the preprocessed PDBbind dataset**


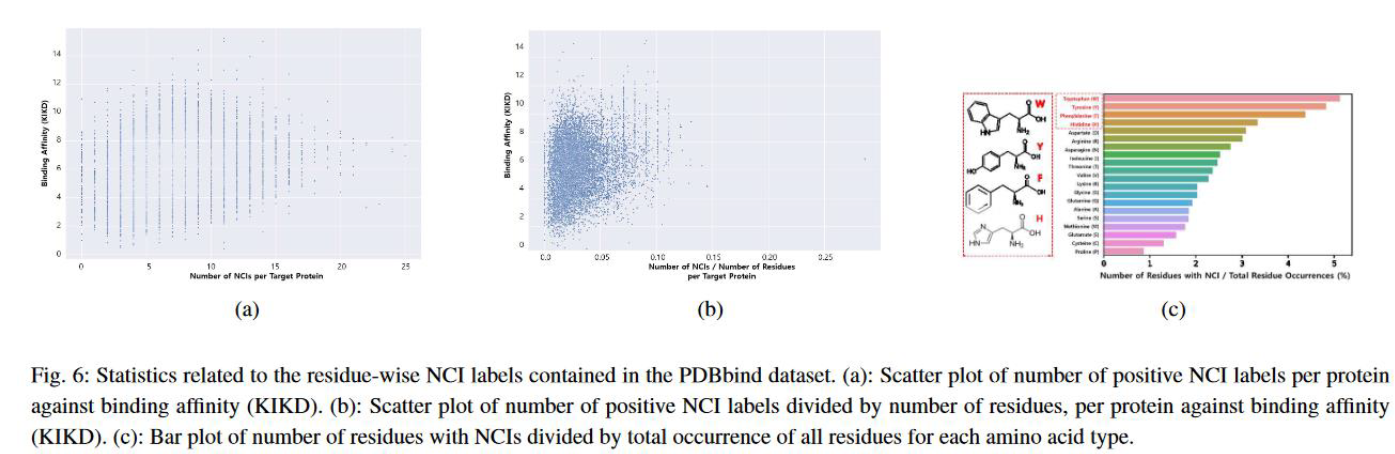


Figure S2. Statistics related to the residue-wise NCI labels contained in the PDBbind dataset. (a): Scatter plot of number of positive NCI labels per protein against binding affinity (KIKD). (b): Scatter plot of number of positive NCI labels divided by number of residues, per protein against binding affinity (KIKD). (c): Bar plot of number of residues with NCIs div

**3. Illustrative description of the auxiliary loss objective based on attention regularization guided by NCIs.**


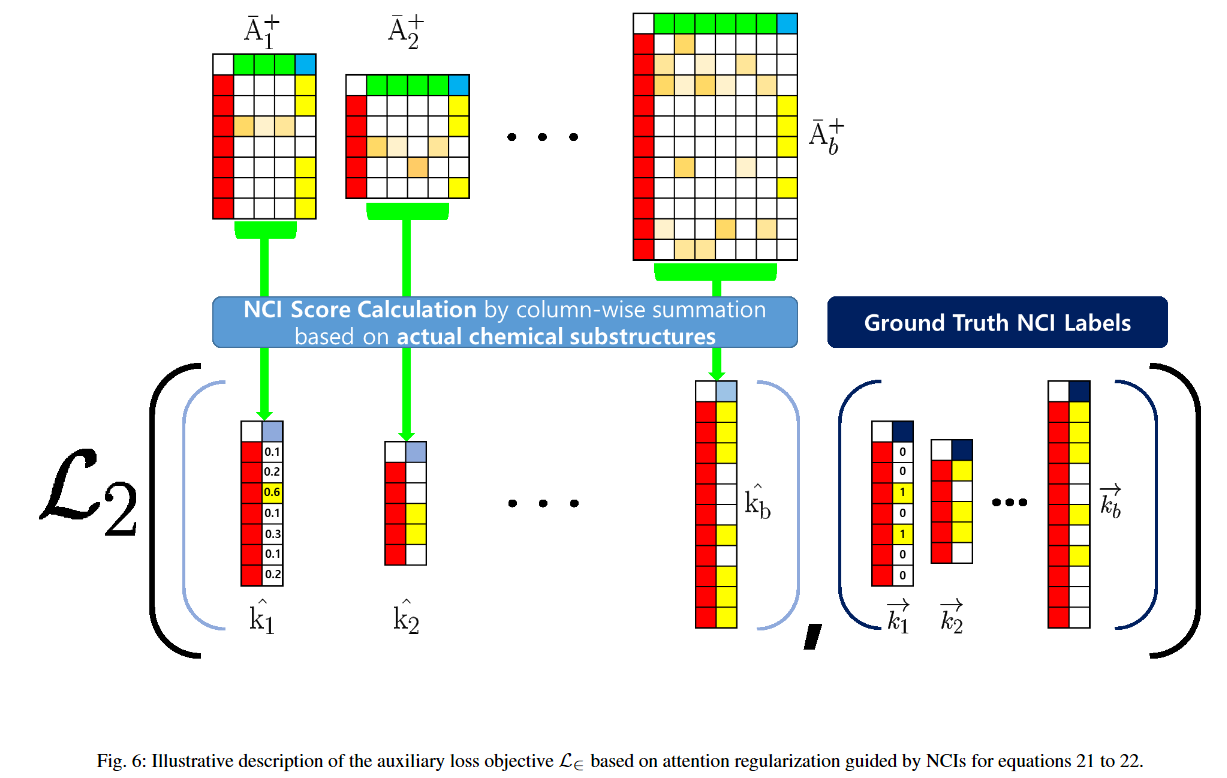


Figure S3. Illustrative description of the auxiliary loss objective based on attention regularization guided by NCIs. For each 2D matrix of attention weights based on its corresponding protein-ligand input pair, the residue-wise (row-wise) NCI scores are calculated by column-wise summation on actual chemical substructures excluding the last column (pseudo-substructure). The loss objective computes Cross Entropy between list of NCI scores and its ground truth NCI labels.

**4. Attention Map comparison between *ArkDTA* and *ArkDTA* (Remove L2)**


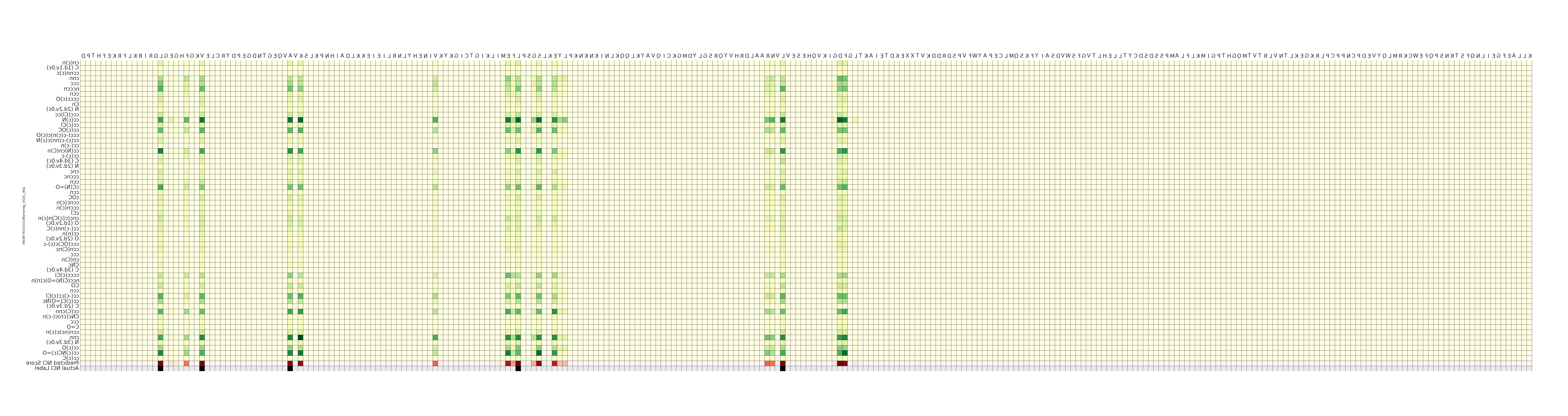


**
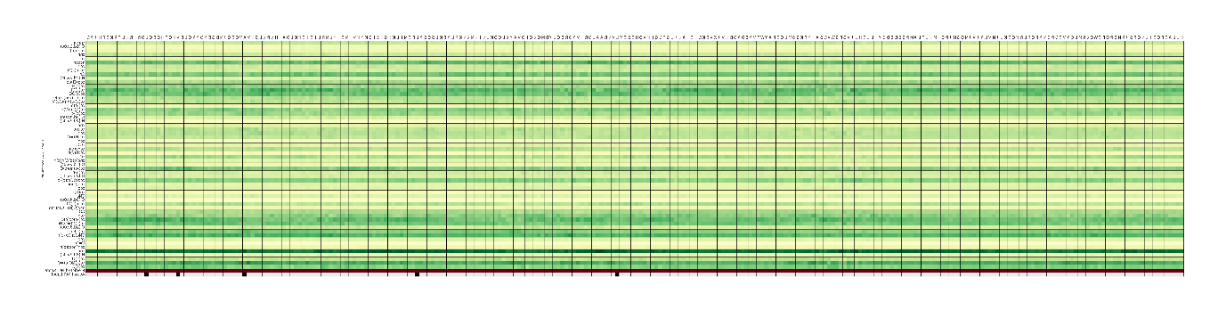
**

Figure S4. Attention map comparison for 6n77 between ArkDTA (top) and its ablated version (bottom) where it the loss coefficient for the auxiliary loss objective (attention regularization guided by NCIs).

**5. Full-sized Attention Maps**


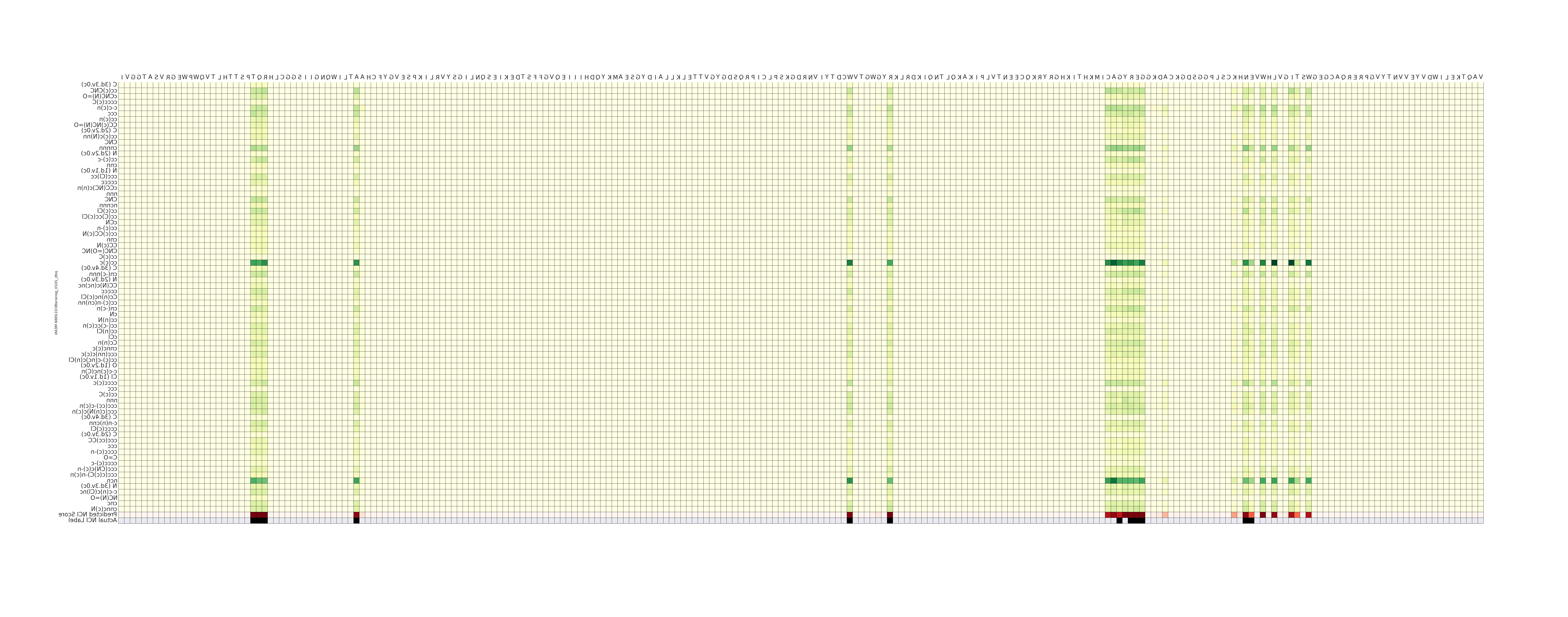

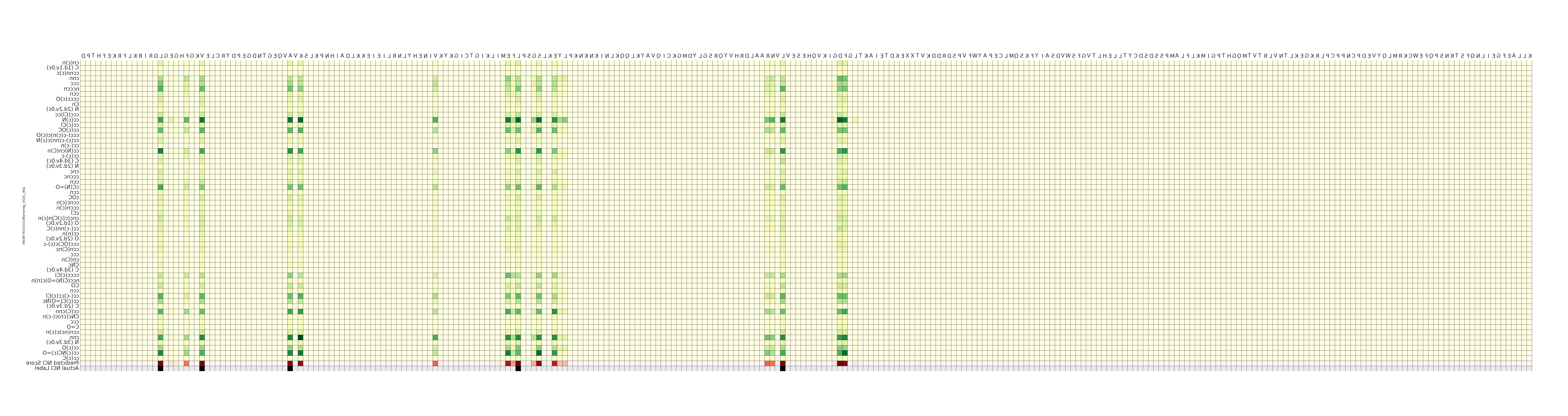

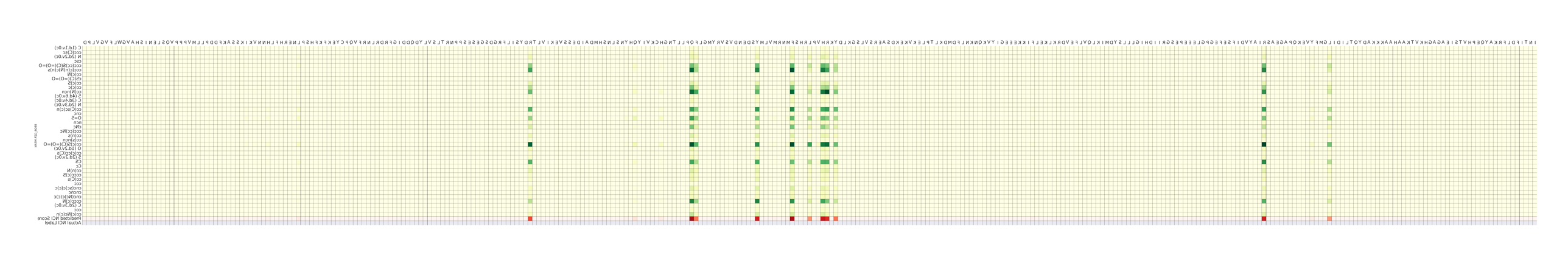


Figure S5. Full-sized attention maps for case studies 4x6n, 6n77, 8bdq (top to bottom)

**6. Visualization results on three more protein-ligand pair examples**


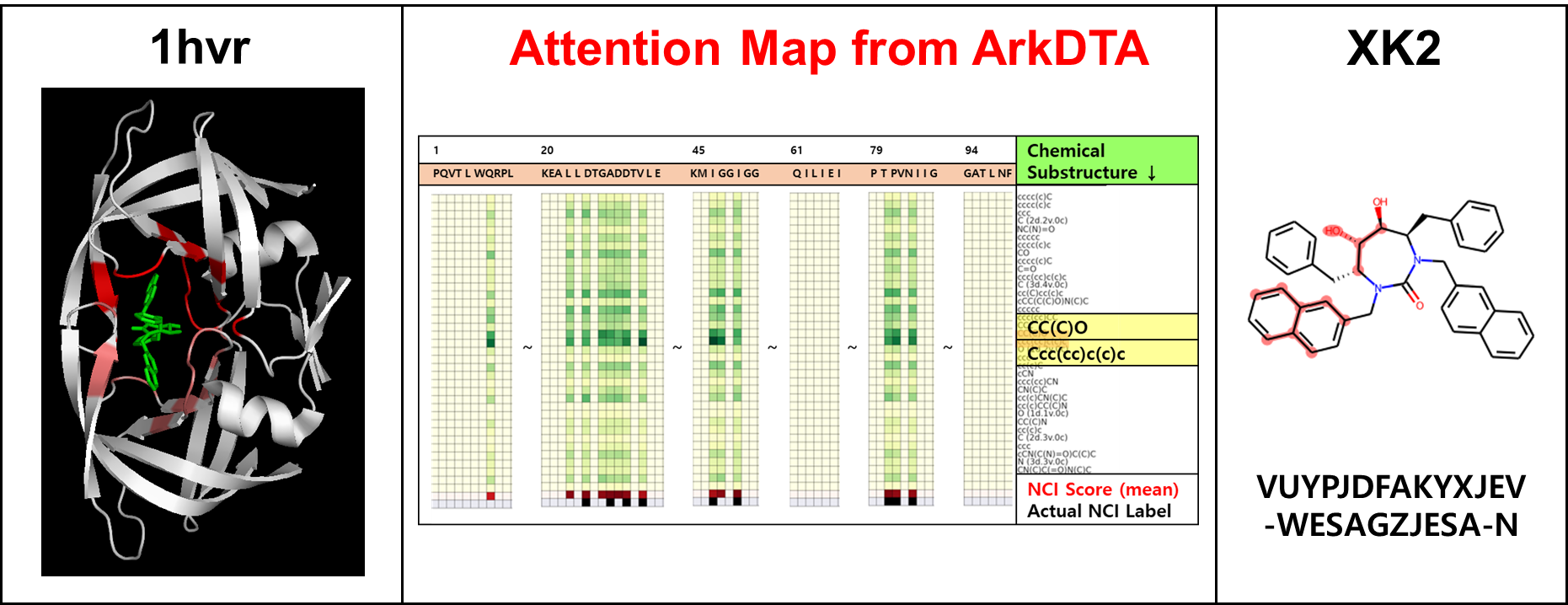


1. **Seen Protein & Unseen Ligand Binding Complex (1hvr, XK2)**


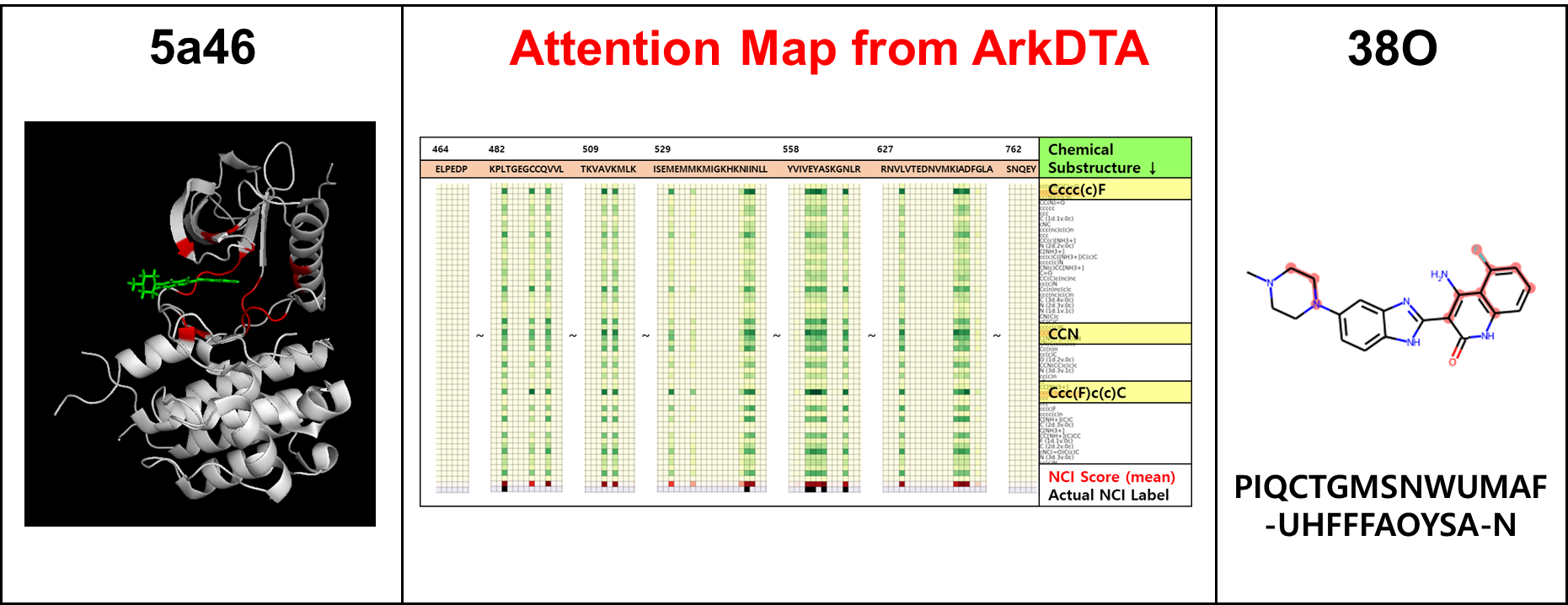


1. **Unseen Protein-Ligand Binding Complex (5a46, 38O)**

**
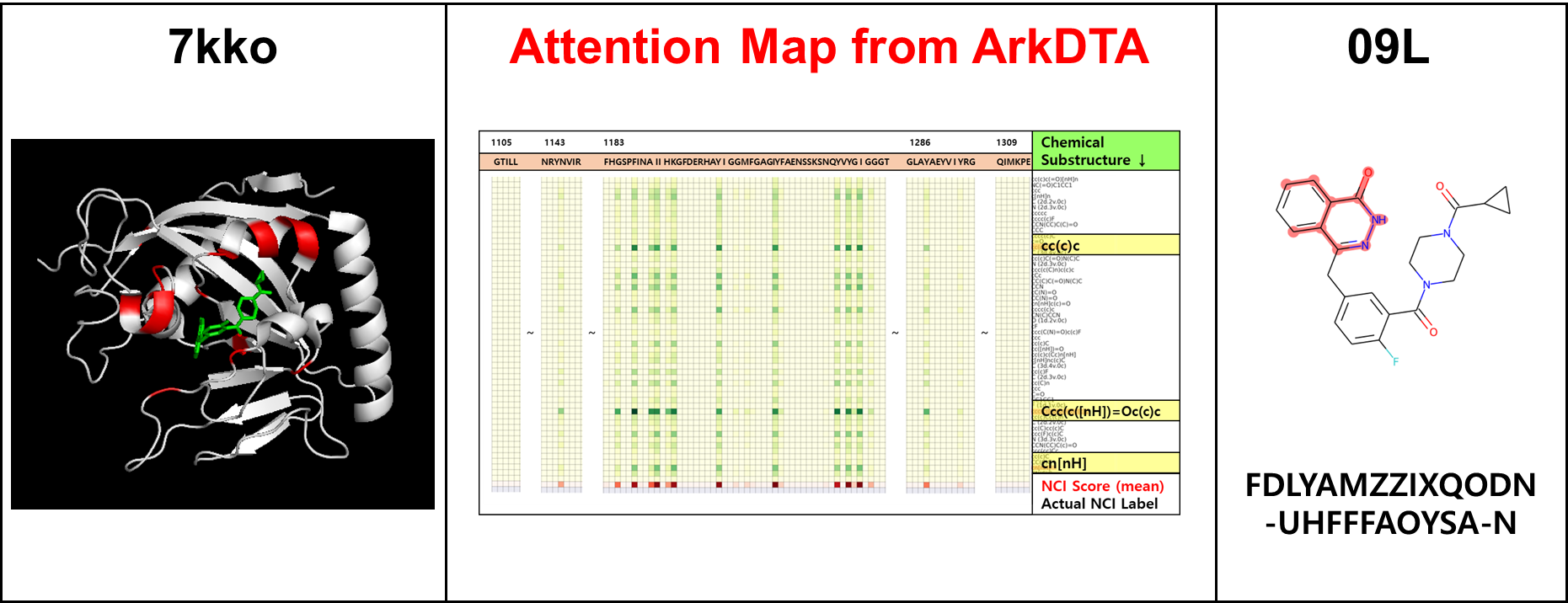
**

1. **Out-of-Dataset Binding Complex (7kko, 09L)**

Figure S6. Visualization results on three more protein-ligand pairs. (a) shows the results performed on a binding complex structure of HIV-1 protease with the inhibitor ligand. (b) shows the results performed on a binding complex structure of FGFR1 kinase with Dovitinib. (c) shows the results performed on a binding complex structure of catalytic domain of tankyrase 1 with Olaparib.
